# The GTPase-Activating Protein Rga1 Interacts with Rho3 GTPase and May Regulate Its Function in Polarized Growth in Budding Yeast

**DOI:** 10.1371/journal.pone.0123326

**Published:** 2015-04-10

**Authors:** Fei He, Wen-Chao Nie, Zongtian Tong, Si-Min Yuan, Ting Gong, Yuan Liao, Erfei Bi, Xiang-Dong Gao

**Affiliations:** 1 Department of Microbiology, College of Life Sciences, Wuhan University, Wuhan, China; 2 Hubei Provincial Cooperative Innovation Center of Industrial Fermentation, Wuhan, China; 3 Department of Cell and Developmental Biology, University of Pennsylvania Perelman School of Medicine, Philadelphia, Pennsylvania, United States of America; Institute of Biology Valrose, FRANCE

## Abstract

In budding yeast, Rga1 negatively regulates the Rho GTPase Cdc42 by acting as a GTPase-activating protein (GAP) for Cdc42. To gain insight into the function and regulation of Rga1, we overexpressed Rga1 and an N-terminally truncated Rga1-C538 (a.a. 538-1007) segment. Overexpression of Rga1-C538 but not full-length Rga1 severely impaired growth and cell morphology in wild-type cells. We show that Rga1 is phosphorylated during the cell cycle. The lack of phenotype for full-length Rga1 upon overexpression may result from a negative regulation by G1-specific Pho85, a cyclin-dependent kinase (CDK). From a high-copy suppressor screen, we isolated *RHO3*, *SEC9*, *SEC1*, *SSO1*, *SSO2*, and *SRO7*, genes involved in exocytosis, as suppressors of the growth defect caused by Rga1-C538 overexpression. Moreover, we detected that Rga1 interacts with Rho3 in two-hybrid and bimolecular fluorescence complementation (BiFC) assays. Rga1 preferentially interacts with the GTP-bound form of Rho3 and the interaction requires the GAP domain and additional sequence upstream of the GAP domain. Our data suggest that the interaction of Rga1 with Rho3 may regulate Rho3’s function in polarized bud growth.

## Introduction

Rho-family GTPases in eukaryotic cells regulate cytoskeletal rearrangement, vesicular trafficking, and cell cycle progression [1,2]. They act as molecular switches, cycling between the GTP- and GDP-bound states. Only in the GTP-bound form can they bind to downstream effectors to transduce signals. The cycling of Rho GTPases requires the guanine nucleotide exchange factors (GEFs) and GTPase-activating proteins (GAPs). GEFs catalyze the exchange of GDP for GTP of Rho proteins, leading to their activation. GAPs promote the intrinsic GTP-hydrolyzing activity of Rho proteins, thereby leading to their rapid conversion to the inactive GDP-bound state [3].

There are six Rho GTPases in the budding yeast *Saccharomyces cerevisiae*: Cdc42 and Rho1 to Rho5 [4]. Cdc42, Rho1, Rho3, and Rho4 are important for polarized organization of the cytoskeleton. This function requires their interaction with the formins, Bni1 or Bnr1 [5]. Cdc42 is critical for bud emergence because temperature-sensitive *cdc42* mutants became large and round without a bud at restrictive temperature [6]. Rho3 and Rho4 are important for the maintenance of bud growth after bud emergence since *rho3*Δ *rho4*Δ cells often lysed at the small-budded stage with a large and round morphology [7]. Besides their roles in actin organization, Cdc42 and Rho3 also have a direct role in exocytosis (polarized secretion). *cdc42-6* and *rho3-V51* mutants accumulated post-Golgi secretory vesicles while the actin cytoskeleton was still polarized [8,9]. The function of Rho3 in exocytosis is thought to be mediated by two downstream effectors, Myo2 (myosin V, involved in the transport of vesicles) and Exo70 (exocyst component, implicated in the docking of vesicles on the plasma membrane) [10,11], while the role of Cdc42 in exocytosis is thought to be mediated by its effectors, Sec3 and Exo70, two exocyst components [11,12]. It is interesting to note that the functions of Cdc42 and Rho3 overlap to some extent. High-copy *RHO3* rescued the growth defect of *cdc42-6* mutant whereas high-copy *CDC42* and *BEM1* rescued the lethality of *rho3*Δ *rho4*Δ cells [7,9].

Cdc42 has four GAPs: Rga1/Dbm1, Rga2, Bem2, and Bem3 [4], all of which are large proteins with RhoGAP domain at their C termini. Rga1 acts specifically on Cdc42. It interacts with the GTP-bound form of Cdc42 in two-hybrid assay [13,14]. Recombinant GAP domain of Rga1 stimulated GTP hydrolysis by Cdc42 [13,15]. Rga2 shares high sequence similarity with Rga1 and may also act specifically on Cdc42 [13]. Bem2 is a GAP for both Cdc42 and Rho1 [16,17]. Bem3 could also act on Rho1 *in vitro*, *albeit* weakly than on Cdc42 [18,19].

The physiological role of Rga1 appears to down-regulate Cdc42 activity since high-copy *RGA1* impaired the growth of temperature-sensitive *cdc42-1* mutant at 35°C while *rga1*Δ restored growth to the temperature-sensitive *cdc24-H* mutant (Cdc24 is a Cdc42GEF) [14]. Rga1 shares a redundant function with Rga2 and Bem3 in Cdc42-controlled conversion of a septin “cloud” into a septin ring at the presumptive bud site [20]. However, Rga1 appears to have certain cellular function not redundant with Rga2 and Bem3. We previously reported that Rga1 plays a critical role in the elimination of active Cdc42-GTP at the bud neck after cytokinesis. This function is crucial for proper bud-site selection at the beginning of the next cell cycle, as *rga1*Δ cells, unlike *rga2*Δ or *bem3*Δ cells, budded within the old budding site [21]. Rga1 also appears to act much more potently than Rga2 and Bem3 in the negative regulation of the pheromone response pathway [13]. Apart from these, whether or not Rga1 may have other unique cellular functions is not clear.

Here, we show that a high dose of N-terminally truncated Rga1 impairs polarized bud growth and this defect could be rescued by high-copy *RHO3* and genes encoding components of the exocytic apparatus but not by high-copy *CDC42*. In addition, Rga1 interacts with Rho3 *in vivo*. These results suggest that Rga1 may normally engage in the regulation of Rho3 in polarized growth. We also show that Rga1 is phosphorylated in the cells and the phosphorylation event negatively regulates its function.

## Results

### Overexpression of the C-terminal region of Rga1 impairs polarized bud growth

**Fig 1:**
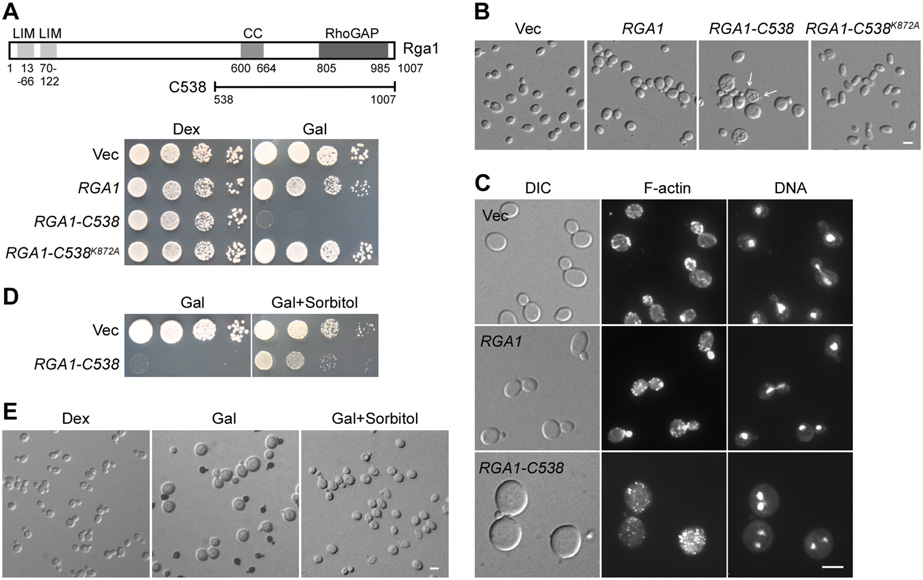
Phenotypes of yeast cells overexpressing Rga1-C538. **(A)** Cells of strain YEF473A carrying integrated pEGKT306 (Vec), pEGKT306-RGA1, pEGKT306-RGA1-C538, and pEGKT306-RGA1-C538^K872A^ plasmids were spotted on SC-Ura (Dex) and SRG-Ura (Gal) plates at 1:10 serial dilution and incubated at 30°C for 4 days. Upper panel, schematic representation of Rga1 and Rga1-C538 segement. CC, coiled-coil. **(B)** DIC images of cells as in (A) grown on SRG-Ura plate for 16 hr at 30°C. **(C)** F-actin and DNA staining of cells grown on SRG-Ura plate for 16 hr at 30°C. **(D)** YEF473A cells carrying pEGKT306 (Vec) and pEGKT306-RGA1-C538 plasmids were grown on SRG-Ura (Gal) and SRG-Ura+1 M Sorbitol plates at 30°C. Pictures were taken after 4 days. **(E)** Methylene blue staining of YEF473A cells carrying integrated pEGKT306-RGA1-C538 grown on SC-Ura, SRG-Ura and SRG-Ura+1 M Sorbitol plates for 12 hr at 30°C. Bars, 5 μm.

Because of the functional redundancy between Rga1 and three other Cdc42GAPs (Rga2, Bem2, and Bem3), it is difficult to determine precisely how Rga1 is involved in the control of Cdc42-regulated functions. Here, we tried to examine Rga1’s cellular function by overexpressing Rga1. In theory, overexpression of Rga1 would reduce the activity of Cdc42, generating a *cdc42* mutant-like loss-of-polarity phenotype. Surprisingly, we found that overexpression of full-length Rga1 in wild-type cells under the control of a galactose-inducible promoter did not dramatically impair growth (Fig 1A), or cell morphology (Fig 1B). We then overexpressed an N-terminally truncated Rga1-C538 (a.a. 538–1007) segment (Fig 1A), a presumably dominant-active mutant based on its ability to suppress the growth and morphological defects of temperature-sensitive *bem2* mutants [22]. Interestingly, overexpression of Rga1-C538 was toxic to the cells. No colonies were formed on SRG-Ura agar medium within 4 days at 30°C (Fig 1A). Cell morphology was also affected.

Rga1-C538 overexpression caused a loss-of-polarity phenotype. Microscopic examination of cells induced for Rga1-C538 overexpression for 16 hours at 30°C revealed that 63% of the cells examined (*n* = 238) became large and round, indicative of a loss of cell polarity. Among the large and round cells, 58% (*n* = 263) were unbudded. The remaining 42% were budded, but usually had a small or tiny bud (Fig 1B). Some cells even carried two or more buds (mostly two) on the same mother cell (Fig 1B, see arrows). All the budded cells examined (*n* = 72) displayed a depolarized actin cytoskeleton as actin patches were no longer enriched in the buds. Actin cables were barely visible (Fig 1C, bottom panel). These findings suggest that Rga1-C538 overexpression caused a defect in polarized bud growth. Staining of nuclear DNA revealed that 40% of large and round cells (*n* = 198) contained two or more nuclei in the mother cell (Fig 1C, bottom panel), which was not seen in wild-type control cells, nor in cells overexpressing full-length Rga1. In the multinucleated population of large and round cells, 36% (*n* = 224) were unbudded. The remaining 64% carried at least one bud. This observation indicates that nuclear segregation was defective.

The growth defect caused by Rga1-C538 overproduction could be effectively suppressed by the addition of 1 M sorbitol, an osmolarity-stabilizing agent, to the culture medium (Fig 1D). The large and round cell morphology was also partially suppressed, though most cells examined (59%; *n* = 276) were still markedly bigger than control cells (Fig 1E). This observation suggests that cell wall may be defective in cells overexpressing Rga1-C538. In support of this view, we observed that after 12 hours’ induction for Rga1-C538 overexpression, 30% of cells were dead as indicated by staining with methylene blue, a vital staining dye that selectively stains dead cells blue, but not live cells [23] (Fig 1E). Interestingly, among the dead cells, nearly all (92%; *n* = 304) had a normal morphology and 73% (*n* = 304) were small-budded. For comparison, only 16% of live cells (*n* = 258) were small-budded. Moreover, after the addition of sorbitol to the medium, the death of normal looking cells was almost completely suppressed (3% dead cells; *n* = 245) (Fig 1E). These results suggest that Rga1-C538 overexpression may affect cell wall integrity and cause cell lysis at the small-budded stage.

The defect in polarized bud growth caused by Rga1-C538 overexpression seems to depend on an interaction with Cdc42 because overexpression of the Rga1-C538^K872A^ mutant neither impaired growth (Fig 1A), nor enlarged the cells (Fig 1B). It has been shown that the K872A mutation in Rga1 not only eliminated its GAP activity but also its interaction with Cdc42 [15]. This result implies that Cdc42 may be the direct target of Rga1-C538 action. An increase of Rga1 activity may down-regulate Cdc42 activity, causing a loss of cell polarity. The lack of phenotype for full-length Rga1 upon overexpression suggests that the GAP activity of Rga1 may normally be regulated (see next section).

Taken together, our results suggest that an excess of Rga1 GAP activity affects polarized bud growth, causing depolarized growth and cell lysis at the small-budded stage.

### Rga1 may be negatively regulated by phosphorylation

The observation that overexpression of the N-terminally truncated Rga1-C538 segment impaired growth whereas overexpression of full-length Rga1 did not implies that the GAP activity of Rga1 may normally be negatively regulated in the cells. The truncated Rga1 segment may lack this regulation and became hyperactive.

**Fig 2:**
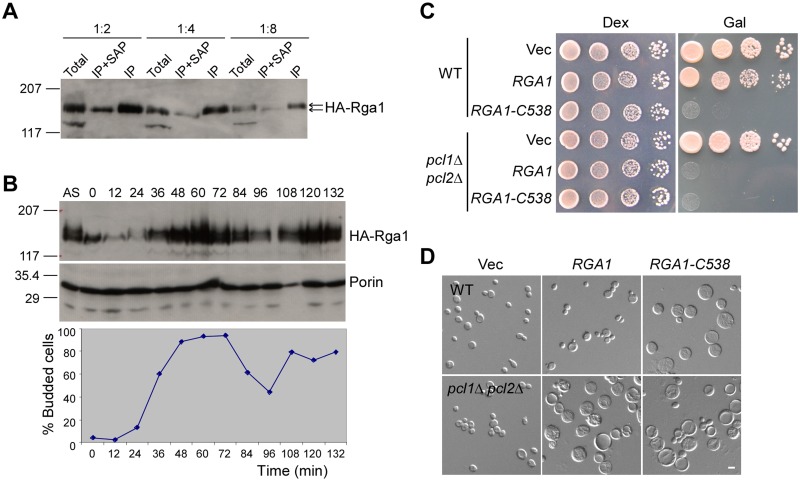
Rga1 may be negatively regulated by phosphorylation. **(A)** Rga1 is phosphorylated during the cell cycle. HA-Rga1 was immunoprecipated from cell lysates of strain YZT52 by anti-HA antibodies conjugated to Sepharose beads. Samples of cell lysates (total), immunoprecipates (IP), and shrimp alkaline phosphatase-treated immunoprecipates (IP+SAP) were diluted and analyzed by immunoblotting using anti-HA antibodies. Molecular weight (kDa) markers were shown. **(B)** Cells of strain YZT52 were synchronized in G1 with α factor (time 0) and then released into fresh medium. HA-Rga1 was analyzed by immunoblotting at indicated time points (min). Porin serves as a loading control. The percentage of budded cells at each time point was determined microscopically. AS, asynchronized culture. **(C)** Cells of strains BY448 (WT) and BY634 (*pcl1*Δ *pcl2*Δ) carrying pEGKT306 (Vec), pEGKT306-RGA1, or pEGKT306-RGA1-C538 were grown on SC-Ura (Dex) and SRG-Ura (Gal) plates at 30°C. Pictures were taken after 4 days. **(D)** DIC images of cells as in (C) were grown on SRG-Ura plate for 16 hr at 30°C. Bar, 5 μm.

HA-tagged HA-Rga1 immunoprecipitated from yeast cell lysate showed a small mobility shift in SDS-PAGE (Fig 2A, see also Fig 2B, compare samples AS and 0 min). This shift can be eliminated by the treatment of a phosphatase (Fig 2A), indicating that Rga1 is phosphorylated in the cells, in agreement with previous reports [24,25]. To monitor the phosphorylation of Rga1 during cell cycle progression, we synchronized the cells at late G1 by α factor. The cells were then released into fresh medium and samples were collected every 12 min for Western blotting. We observed that Rga1’s protein level varied dramatically during the cell cycle. The amount of protein was low at G1 but high at M phase (Fig 2B). Rga1 phosphorylation was prominent at G2 and M phases (Fig 2B).

Rga1 is thought to be phosphorylated by Cdc28-Cln2, a G1-specific CDK (cyclin-dependent kinase) [24] and Cdc28-Clb2, a mitotic CDK [25]. Rga2, the paralogue of Rga1, is known to be negatively regulated by Cdc28 and Pho85, another G1-specific CDK, by phosphorylation [26,27,28]. Because Rga1 shares extensive sequence homology over their entire lengths with Rga2, we asked if Rga1 might also be a target of Pho85 CDK. To test this, we overexpressed full-length Rga1 in *pcl1*Δ *pcl2*Δ cells. Pcl1 and Pcl2 are cyclins that specifically bind to and activate Pho85. We found that overexpression of full-length Rga1 severely inhibited the growth of *pcl1*Δ *pcl2*Δ cells, but not wild-type cells (Fig 2C). *pcl1*Δ *pcl2*Δ cells became large and round, similar to the effect of Rga1-C538 overexpression (Fig 2D).

There are 15 consensus CDK phosphorylation sites (S/T-P) in Rga1. Among them, only one site locates within the RhoGAP domain. The remaining 14 sites locate in the middle region between the two LIM domains and the RhoGAP domain. Because 10 out of 15 CDK consensus sites locate in the region a.a. 1–537, which is missing in the dominant-active Rga1-C538 segment, the N-terminal half of the protein may contain the key phosphorylation sites. Interestingly, Thr 278 locates in the only full consensus CDK phosphorylation site (S/T-P-X-K/R) in Rga1. It could be a major CDK phosphorylation site. However, we found that Rga1^T278A^ mutant that mimics the unphosphorylated form did not impair growth in wild-type cells upon overexpression (data not shown). It is likely that the phosphorylation of multiple sites may be required for the negative regulation, like the case of Rga2, which involves in the phosphosphorylation of at least 8 sites [28].

Together, our results indicate that Rga1 is a phosphoprotein. Rga1 may be an important substrate of the G1-specific Pho85 CDK and the phosphorylation of Rga1 by CDK may negatively regulate Rga1 activity to ensure proper Cdc42 activation during bud emergence.

### An excess of Rga1 activity may impair exocytosis

**Fig 3:**
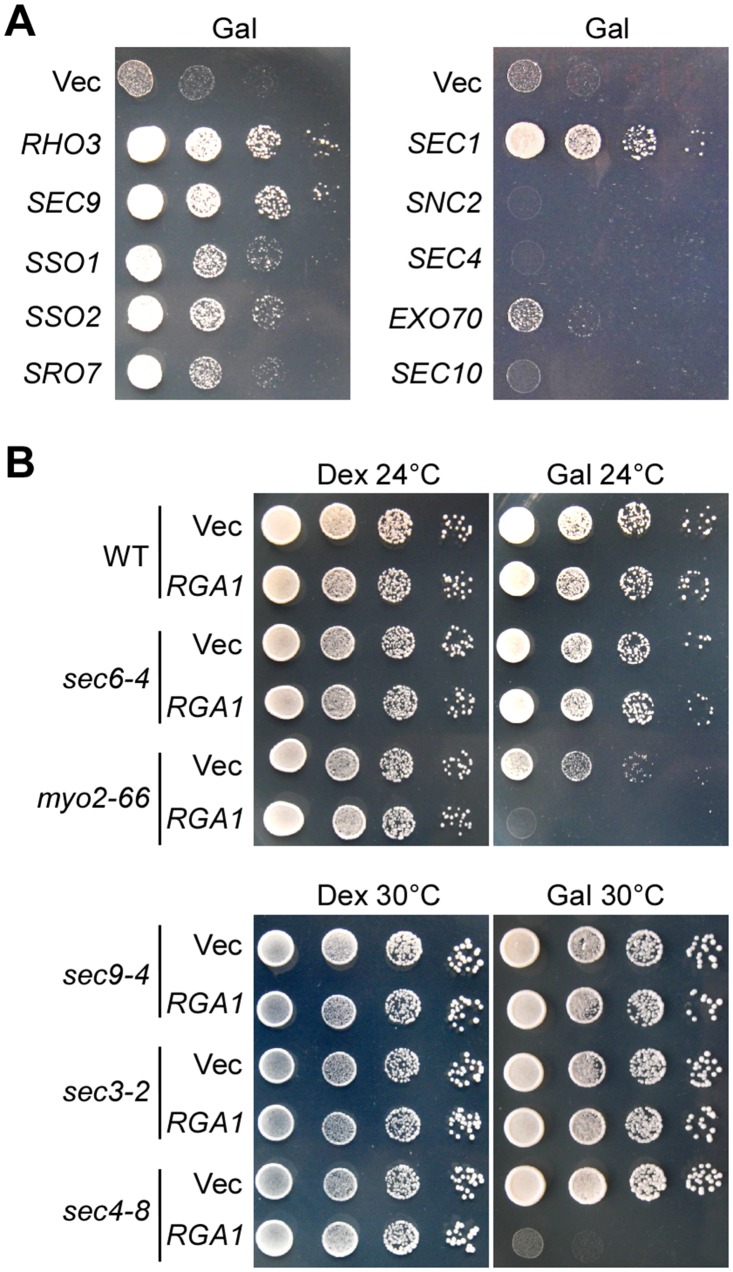
Excess Rga1 activity may impair exocytosis. **(A)** Suppression of the toxicity of Rga1-C overexpression by high-copy suppressors. YEF473A cells carrying integrated pEGKT306-RGA1-C538 plasmid along with replicative YEp13 (Vec), YEp13-RHO3, YEp13-SEC9, YEp13-SSO1, YEp13-SSO2, YEp13-SRO7, YEp13-SEC1, YEp181-SEC4 plasmids and cells carrying integrated pEGKT305-RGA1-C538 along with replicative pRS426-SNC2, pRS426-EXO70, and pRS426-SEC10 plasmids were grown on SRG-Leu-Ura plates at 30°C. Pictures were taken after 4 days. **(B)** Full-length Rga1 overexpression reduced the growth of *sec4-8* and *myo2-66* mutants. Cells of strain YEF473A (wild type, WT), JGY32B (*sec3-2*), JGY29B (*sec4-8*), JGY30A (*sec6-4*), JGY31B (*sec9-4*), and SLY33 (*myo2-66*) carrying pGAL4 (Vec) and pGAL4-RGA1 (*GAL-RGA1*) were grown on SC-Leu (Dex) and SRG-Leu (Gal) plates at 30°C or 24°C. Pictures were taken after 4 days.

To gain insight into how Rga1-C overexpression affected cell growth, we performed a high-copy suppressor screen looking for genes that, when mildly overexpressed, could rescue the lethality caused by Rga1-C538 overproduction (see details of the screen in Materials and Methods). *RHO3*, *SEC9*, *SSO1*, *SSO2*, *SRO7*, and *SEC1* genes were isolated from the screen. Among them, *RHO3*, *SEC9*, and *SEC1* showed the strongest suppression for the growth defect (Fig 3A). It is interesting to note that all the protein products encoded by these genes are implicated in exocytosis. Rho3 controls the transport and fusion of secretory vesicles into the growing bud. Sec9, Sso1, and Sso2 are plasma membrane t-SNAREs that participate in the fusion of secretory vesicles with the plasma membrane [29–31]. Sro7 interacts with both Sec9 and Exo84, a component of the exocyst, and functions in the docking and fusion of post-Golgi vesicles with the plasma membrane [32,33]. Sec1 binds to the assembled SNARE complex and is involved in the activation of membrane fusion [34,35]. The observation that these genes could suppress the lethality caused by Rga1-C538 overexpression suggests that excess Rga1-C538 may impair exocytosis.

High-copy *SEC10*, which encodes a component of exocyst [36], did not suppress the lethality. The lethality was not rescued by high-copy *SEC4* or *SNC2*, either (Fig 3A). Sec4 is a Rab GTPase that plays a crucial role in vesicular transport [37,38]. Snc2 is the v-SNARE located on secretory vesicles [39]. Interestingly, high-copy *EXO70* weakly suppressed the lethality caused by Rga1-C538 overproduction (Fig 3A). Exo70 is another component of exocyst and is a downstream effector of Rho3 [10,11]. Because a subset of exocytic genes implicated in the vesicle fusion step suppressed the lethality caused by Rga1-C538 overproduction, Rga1-C538 overexpression may affect the vesicle fusion step of exocytosis.

To test whether vesicular transport may also be affected, we overexpressed full-length Rga1 in temperature-sensitive *myo2-66* and several post-Golgi secretory mutants. We found that Rga1 overexpression reduced the growth of *myo2-66* (at 24°C) and *sec4-8* (at 30°C) mutants, but not the growth of *sec3-2*, *sec6-4*, or *sec9-4* mutants (Fig 3B). Because Myo2 (myosin V) and Sec4 are essential for vesicular transport, this result suggests that an excess of Rga1 activity may also affect vesicular transport.

Together, our results suggest that excess Rga1 activity may impair polarized growth by affecting exocytosis.

### Rho GTPases other than Rho3 did not rescue the lethality caused by Rga1-C538 overexpression

**Fig 4:**
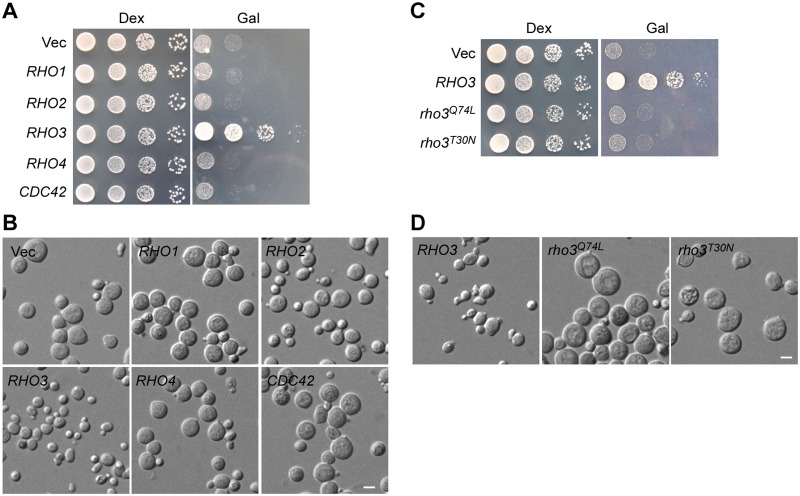
Rho3 suppresses the defect of Rga1-C538 overexpression. **(A)** YEF473A cells carrying integrated pEGKT305-RGA1-C538 overexpression plasmid along with replicative YEp24 (Vec), YEp24-RHO1, YEp24-RHO2, YEp24-RHO3, pRS426-RHO4, and YEp24-CDC42 were grown on SC-Leu-Ura (Dex) and SRG-Leu-Ura (Gal) plates at 30°C. Pictures were taken after 4 days. **(B)** DIC images of cells as in (A) grown on SRG-Leu-Ura plate for 20 hr at 30°C. **(C)** YEF473A cells carrying pEGKT305-RGA1-C538 along with pRS426 (Vec), pRS426-RHO3, pRS426-RHO3^Q74L^, and pRS426-RHO3^T30N^ were grown on SC-Leu-Ura (Dex) and SRG-Leu-Ura (Gal) plates at 30°C. Pictures were taken after 4 days. **(D)** DIC images of cells as in (C) grown on SRG-Leu-Ura plate for 20 hr at 30°C. Bars, 5 μm.

Cdc42 is a known *in vivo* target of Rga1. Rga1-C538 overexpression would in theory inactivate Cdc42, causing a growth defect. Surprisingly, we isolated *RHO3* but not *CDC42* from the high-copy suppressor screen. An assay using high-copy *CDC42* showed that a high dose of Cdc42 indeed did not suppress the growth defect caused by Rga1-C538 overexpression (Fig 4A). The morphology of cells was not suppressed, either (Fig 4B). In contrast, high-copy *RHO3* efficiently suppressed the growth defect (Fig 4A). The morphology defect was also partially suppressed by *RHO3* as the percentage of large and round cells was 31% (*n* = 233) after 20 hours’ induction for Rga1-C538 overexpression, lower than that of control cells (66%; *n* = 229). Dead cells (indicated by methylene blue staining) was just 8% (*n* = 278), significantly lower than that of control cells (27%; *n* = 215).

Among the six Rho GTPases, Rho4 is the closest to Rho3 with respect to cellular functions although they do not share a high degree of amino acid sequence similarity [40]. We found that high-copy *RHO4* did not suppress the growth and cell morphology defect (Fig 4A and 4B). High-copy *RHO1* or *RHO2* also failed to suppress the defects (Fig 4A and 4B). The specific suppression by Rho3 but not by other Rho GTPases suggests that Rho3 might be another target of Rga1 *in vivo*. Rga1-C538 overexpression may down-regulate Rho3 together with Cdc42, leading to a growth defect. In support of this idea, we found that Rho3’s suppression activity requires its cycling between the GTP- and GDP-bound states because constitutively active *rho3* ^*Q74L*^ or inactive *rho3* ^*T30N*^ mutants did not suppress either the growth or cell morphology defect caused by Rga1-C538 overexpression (Fig 4C and 4D).

### Rga1 interacts with Rho3 *in vivo*

**Fig 5:**
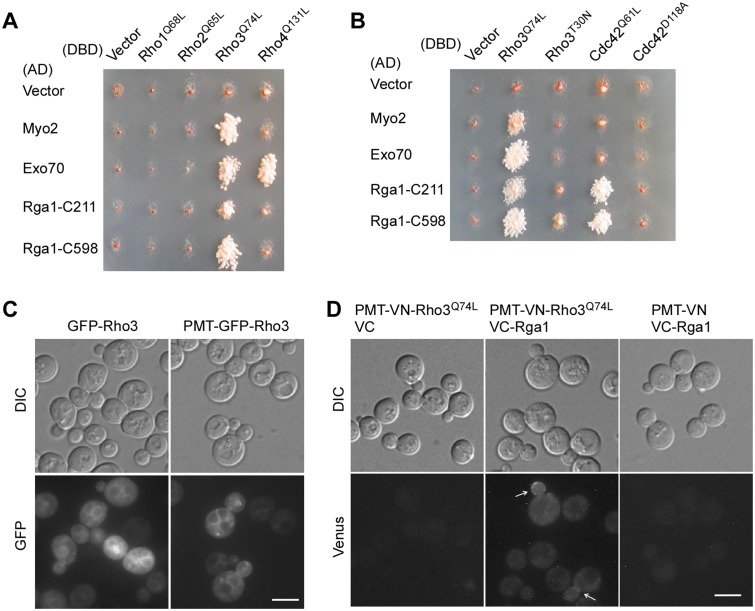
Rga1 interacts with Rho3 *in vivo*. **(A)** Two-hybrid assay of the interaction between Rga1 and four Rho GTPases. The assay was performed as described in *Materials and Methods*. Cells were grown on SC-Leu-Trp-Ade plate. Picture was taken after 4 days at 30°C. Growth indicates interaction between the DBD and AD fusion proteins. **(B)** Two-hybrid assay of the interaction of Rga1-C211 and Rga1-C598 with Rho3 and Cdc42. Cells were grown on SC-Leu-Ura-Ade plate. Picture was taken after incubation at 30°C for 3 days. **(C)** GFP-Rho3 and PMT-GFP-Rho3 localization. YEF473A cells carrying pUG36-RHO3 (GFP-Rho3) and pUG36-PMT-RHO3 (PMT-GFP-Rho3) were visualized for GFP fluorescence. **(D)** BiFC assay between Rga1 and Rho3. Cells of strain JGY2653 (*rho3*Δ) carrying pVN1-PMT-RHO3^Q74L^/pVC1, pVN1-PMT-RHO3^Q74L^/pVC1-RGA1, and pVN1/pVC1-RGA1 pairs were grown in SC-Ura-His medium. Green fluorescence was examined by fluorescence microscopy. Bars, 5 μm.

**Fig 6:**
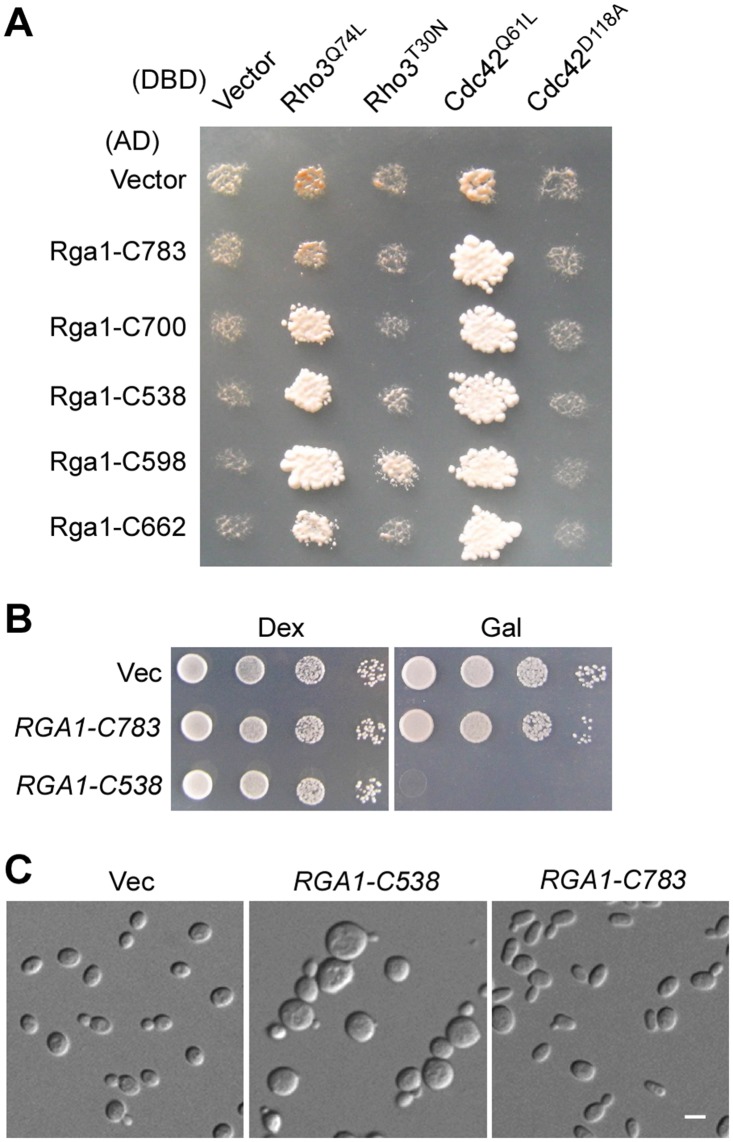
Interaction of Rga1 with Rho3 may require additional sequence upstream of Rga1’s RhoGAP domain. **(A)** Two-hybrid assay of the interaction of Rga1-C segments with Rho3 and Cdc42. Cells were grown on SC-Leu-Ura-Ade plate. Picture was taken after 3 days at 30°C. Prey plasmids pGAD-C1 (AD vector) and pGAD-RGA1-C segments were used. **(B)** Cells of strain YEF473A carrying plasmids pEGKT306 (Vec), pEGKT306-RGA1-C538, and pEGKT306-RGA1-C783 were grown on SC-Ura (Dex) and SRG-Ura (Gal) plates for 4 days at 30°C. **(C)** DIC images of cells as in (B) grown on SRG-Ura plate for 16 hr at 30°C.

In a two-hybrid screen searching for proteins that interacts with Rho3^Q74L^, the constitutively active mutant of Rho3, we isolated two N-terminally truncated Rga1 segments, Rga1-C211 (a.a. 211–1007) and Rga1-C598 (a.a. 598–1007), which contain the RhoGAP domain and the predicted coiled-coil motif but lack the N-terminal LIM domains, along with two known Rho3-interacting proteins, Myo2 and Exo70 [10]. Both Rga1-C211 and Rga1-C598 interacted specifically with Rho3 but not with Rho1, Rho2, or Rho4 in two-hybrid assay (Fig 5A). The interaction between Rga1 and Rho3 is surprising because Rga1 is thought to be a GAP specific for Cdc42. As expected, both Rga1-C211 and Rga1-C598 interacted with Cdc42 in two-hybrid assay. They specifically interacted with Cdc42^Q61L^ (GTP-bound form) but not with Cdc42^D118A^ (GDP-bound or nucleotide-free form) (Fig 5B). Rga1-C211 and Rga1-C598 also interacted efficiently with Rho3^Q74L^ (GTP-bound form). Rga1-C598 also interacted with Rho3^T30N^ (GDP-bound form) albeit with reduced efficiency (Fig 5B, also see Fig 6). Overall, Rga1-C211 and Rga1-C598 interacted preferentially with Rho3^Q74L^ but not with Rho3^T30N^.

Does Rga1 normally interact with Rho3 *in vivo*? To answer this question, we performed a bimolecular fluorescence complementation (BiFC) assay using full-length Rga1 and Rho3^Q74L^. Rho3 is palmitoylated at the cysteine 5 residue at the N-terminus [41]. Moreover, palmitoylation is important for Rho3’s localization to the plasma membrane [42]. We observed that GFP-Rho3 fusion protein rarely localized to the plasma membrane (Fig 5C), presumably due to the lack of palmitoylation. We then added the region a.a. 1–11 of Rho3 to the N-terminus of GFP-Rho3 and detected the plasma membrane localization of Rho3 (Fig 5C, see PMT-GFP-Rho3). In the BiFC assay, we thus used PMT-Venus-N-Rho3^Q74L^ (Venus is a GFP variant). We found that PMT-Venus-N-Rho3Q74L interacted with Venus-C-Rga1 at the bud cortex and bud neck (Fig 5D, see arrows), locations where Rga1 localized [20].

Taken together, our result showed that Rga1 interacts with Rho3 and preferentially with the active GTP-bound form of Rho3.

### Additional sequence upstream of the RhoGAP domain of Rga1 is required for efficient interaction with Rho3

To locate the region in Rga1 that interacts with Rho3, we generated several Rga1-C constructs that share intact C-terminus and examined their interaction with Rho3 and Cdc42 using two-hybrid assay. We found that Rga1-C662 (a.a. 662–1007) and Rga1-C700 (a.a. 700–1007) all interacted with Rho3^Q74L^. In contrast, the shorter segment, Rga1-C783 (a.a. 783–1007), did not interact with Rho3^Q74L^ (Fig 6A). All these Rga1-C segments interacted with Cdc42^Q61L^ efficiently like Rga1-C598 did. This result suggests that the interaction of Rga1 with Rho3 may require both the GAP domain and the region a.a. 700–783 upstream of the GAP domain. Interestingly, we observed that overexpression of Rga1-C783, which no longer interacts with Rho3 in two-hybrid assay, did not impair cell growth (Fig 6B). Nor did it cause a loss of cell polarity (Fig 6C). This observation supports the idea that Rga1 may regulate Rho3 in polarized growth.

## Discussion

### Rga1 may be a novel GAP for Rho3

The regulation of Rho GTPases requires guanine nucleotide-exchange factors (GEFs) and GTPase-activating proteins (GAPs). Cdc42 have several GAPs [13,16]. In contrast, there is only one GAP, Rgd1, identified so far for Rho3 [43]. Rgd1 also functions as a GAP for Rho4, which has an overlapping function with Rho3. In this study, we identified an interaction between Rho3 and Rga1, a GAP specific for Cdc42. Rga1 preferentially interacts with the GTP-bound form of Rho3 and the interaction requires the GAP domain of Rga1 and additional sequences upstream of the GAP domain. There are two possibilities about the outcome of this interaction. In the first possibility, Rga1 acts as a GAP for Rho3. This interaction would stimulate the intrinsic GTPase activity of Rho3. In the second possibility, Rga1 binds to Rho3-GTP but does not stimulate Rho3’s GTPase activity. Because it is not clear at present whether Rga1 may possess GAP activity towards Rho3 *in vitro*, we are not sure which possibility may be true. Given that high-copy *RHO3* but not *rho3* ^*Q74L*^ suppressed the effect of Rga1-C overexpression, we favor the first possibility.

The Rho3GAP, Rgd1, localizes to the presumptive bud site and the bud tip in small-budded cells [44]. Rga1 shares a localization pattern similar to, though not identical with, that of Rgd1 [20]. In addition, our BiFC assay showed that Rga1 and Rho3 interact at the bud cortex of small-budded cells and at the bud neck in large-budded cells. These observations suggest that Rga1 may function as a GAP for Rho3. Rga1 may play a role in the regulation of both Cdc42 and Rho3 during the cell cycle.

In this study, we showed that Rga1 is phosphorylated during the cell cycle and the activity of Rga1, presumably the GAP activity, is negatively regulated by Pho85 CDK. Rga1 may also be regulated by mitotic Clb2-Cdc28 CDK complex because we detected Rga1 hyperphosphorylation around mitosis. A previous study identified that Rga1 is negatively regulated by the epsins, which function as adaptors in endocytosis. The epsin Ent2 interacts with Rga1 via a region upstream of the GAP domain in Rga1 [45]. These results suggest that the regulation of Rga1 could be rather complex in the cells.

### Rga1 may regulate polarized growth through its interaction with Rho3

A polarized actin cytoskeleton is essential for bud emergence and bud growth. The large, round cell morphology and depolarized actin organization in cells overexpressing Rga1-C suggest that an excess of Rga1 activity impairs actin organization. Because Cdc42 and Rho3 both regulate actin organization and the phenotype of cells with excess Rga1 activity is reminiscent of that of *cdc42* and *rho3*Δ *rho4*Δ mutants [6,7], the functions of Cdc42 and Rho3 in actin organization are likely affected by excess Rga1 activity.

Cdc42 and Rho3 also have a direct role in exocytosis independent of their functions in actin organization. *cdc42-6* and *rho3-V51* mutants exhibited a normal actin cytoskeleton but accumulated secretory vesicles at 25°C and 14°C, respectively [8,9]. Moreover, Cdc42 interacts with Sec3 and Exo70, two exocyst components [11,12], whereas Rho3 interacts with Exo70 and Myo2 (myosin V) [10,11]. All three proteins are involved in exocytosis. We speculate that the toxicity of excess Rga1 activity may be partly due to the down-regulation of both Cdc42 and Rho3 functions in exocytosis. This is supported by the observation that high-copy *SEC9* and *SRO7*, which suppressed the growth defect of both *cdc42-6* and *rho3-V51* mutants [9], also effectively suppressed the toxicity of Rga1-C.

The functions of Cdc42 and Rho3 in exocytosis appear to be partially overlapping because *cdc42-6* is synthetically lethal with *rho3-V51* mutant. In addition, high-copy *RHO3* suppressed the growth defect of *cdc42-6* mutant [9]. Conversely, high-copy *CDC42* suppressed the growth defect of *rho3-V51* mutant [8]. *CDC42* or *BEM1* also suppressed the growth defect of *rho3*Δ *rho4*Δ cells [7]. Despite this, some major differences exist between the functions of Cdc42 and Rho3 in exocytosis. First, Rho3 seems to control both vesicular transport and vesicle fusion while Cdc42 mainly controls vesicle fusion because *rho3-V51* mutant accumulated exocytic vesicles in both the bud and the mother cell at 14°C while *cdc42-6* mutant accumulated vesicles mainly in the bud [8,9]. Moreover, Rho3 interacts with Myo2 whereas Cdc42 does not (Fig 5B). Second, Cdc42 function in exocytosis may be restricted to early stage of bud development whereas Rho3 may be implicated in both early and subsequent stages of bud growth because *cdc42-6* mutant accumulated vesicles only in the small-budded stage [9]. We speculate that the ability of *RHO3* to suppress the toxicity of Rga1-C on high-copy plasmids is likely because Rho3 regulates both the transport and fusion of exocytic vesicles while Cdc42 only regulates vesicle fusion. A higher dose of Rho3 could not only counteract the negative regulation by Rga1, allowing the cells to polarize actin cytoskeleton and deliver secretory vesicles into the bud, it could also replenish Cdc42 at the bud tip in small-budded cells because Cdc42 is known to be delivered to the bud by secretory vesicles [46]. In contrast to this scenario, a higher dose of Cdc42 may only strengthen its own force but not that of Rho3. As a result, down-regulation of Rho3-regulated transport of Cdc42 into the growing bud by excess Rga1 activity may, in turn, dampen the effect of an increase in Cdc42 level. This may explain why high-copy *CDC42* did not suppress the toxicity of Rga1-C.

Rga1 may normally play a role in exocytosis via its interaction with Rho3. By modulating the functions of both Cdc42 and Rho3, Rga1 may coordinate Cdc42 and Rho3 to ensure proper bud growth.

## Materials and Methods

### Strains and genetic methods

Yeast strains used in this study are listed in Table 1. Standard culture media and genetic techniques were used expect where noted. *Escherichia coli* strains DH12S (Life Technologies, Gaithersburg, MD) and DH5α (TaKaRa, Japan) were used as hosts for plasmid manipulation. SRG-Ura medium containing 2% galactose and 1% raffinose was used to overexpress genes driven by the *GAL1* promoter. SC-Ura medium contained 2% dextrose. Oligonucleotide PCR primers were purchased from Sangon Biotech (Shanghai, China).

**Table 1:** Yeast strains used in this study.

Strain	Genotype	Source
YEF473A	a *his3-*Δ*200 leu2-*Δ *1 lys2-801 trp1-*Δ *63 ura3-52*	[51]
YZT52	As YEF473A except *bar1*Δ::*LEU2 rga1*Δ::*HIS3 3HA-RGA1*:*URA3*	This study
JGY29B	α *his3-*Δ *200 leu2-*Δ *1 trp1-*Δ *63 ura3-52 sec4-8*	[52]
JGY30A	a *his3-*Δ *200 leu2-*Δ *1 trp1-*Δ *63 ura3-52 sec6-4*	[52]
JGY31B	α *his3-*Δ *200 leu2-*Δ *1 trp1-*Δ *63 ura3-52 sec9-4*	[52]
JGY32B	α *his3-*Δ *200 leu2-*Δ *1 trp1-*Δ *63 ura3-52 sec3-2*	[52]
SLY33	a *leu2 ura3 myo2-66*	E. Bi
BY448	a *his3 leu2 lys2 trp1 ura3 ade2*	[53]
BY634	a *his3 leu2 lys2 trp1 ura3 ade2 pcl1*Δ::*LEU2 pcl2*Δ::*LYS2*	[53]
pJ69-4α	α *his3-*Δ *200 leu2-3*,*112 trp1-901 ura3-52 gal4*Δ *gal80*Δ *LYS2*::*GAL1-HIS3 GAL2-ADE2 met2*::*GAL7-lacZ*	[49]
pJ69-4A	a *his3-*Δ *200 leu2-3*,*112 trp1-901 ura3-52 gal4*Δ *gal80*Δ *LYS2*::*GAL1-HIS3 GAL2-ADE2 met2*::*GAL7-lacZ*	[49]

### Plasmid construction

For overexpression study, full-length *RGA1* and various *RGA1* segments were amplified by PCR using plasmid YEp181-RGA1 or YEp181-RGA1^K872A^ [20] as templates and inserted into *Hind*III- and *Sal*I-digested pEGKT306 (integrative, *URA3*, *UAS*_*GAL1*_ *-P*_*CYC1*_ *-GST-T*_*CYC1*_) [47], pEGKT305, or pGAL4 vectors. pEGKT305 (integrative, *LEU2*, *UAS*_*GAL1*_ *-P*_*CYC1*_ *-GST-T*_*CYC1*_) was generated by replacing the *Pvu*I fragment of pRS305 containing *lacZ*-*ori*-*Amp* ^*r*^ with *Pvu*I fragment of *GAL1*_*UAS*_ *-P*_*CYC1*_ *-GST-T*_*CYC1*_ *-ori-Amp* ^*r*^ fragment amplified by PCR from pEGKT306. pGAL4 (*CEN*, *LEU2*, *P*_*GAL1*_ *-T*_*CYC1*_) was constructed by replacing the *Pvu*I fragment of pRS315 (*CEN*, *LEU2*) with the *Pvu*I fragment of pGAL2 [48] that contains *P*_*GAL1*_ *-T*_*CYC1*_. pEGKT306- and pEGKT305-based plasmids were linearized by *Nco*I and *Bst*EII for integration at the *ura3-52* and *leu2* loci, respectively.

For high-copy suppression analysis, we used plasmids YEp24-RHO1 (a gift from Dr. Wei Guo), YEp24-RHO2 (a gift from Dr. Patrick Brennwald), YEp24-RHO3 (a gift from Dr. John Moskow), YEp24-CDC42 [47], pRS426-RHO4 [48], YEp181-SEC4, pRS426-SNC2, pRS426-EXO70 and pRS426-SEC10 (gifts from Dr. Wei Guo). pRS426-RHO3 (a gift from Dr. Patrick Brennwald) carries *RHO3* gene in a *Sal*I-*Xho*I fragment. To generate pRS426-RHO3^Q74L^, the *rho3* ^*Q74L*^ mutation was transferred into the *RHO3* gene (including the 1000-bp promoter and 204-bp 3’ untranslated region) by overlapping PCR, this fragment was then inserted into *Sac*I- and *Sal*I-digested pRS426. pRS426-RHO3^T30N^ were constructed by inserting an *Xho*I- and *Cla*I-digested fragment from pRS316-RHO3^T30N^ (a gift from Dr. Patrick Brennwald) into pRS426. YEp181-SEC4 carries *SEC4* in a 1.4-kb *Eco*RI-*Bam*HI fragment subcloned from pRS423-SEC4 (a gift from Dr. Patrick Brennwald).

Yeast two-hybrid bait vector pODB80 (2μ, *TRP1*, *GAL4-BD*) and constructs pODB80-RHO1^Q68L,ΔC^, pODB80-RHO2^Q65L,ΔC^, pODB80-RHO3^Q74L,ΔC^, and pODB80-RHO4^Q131L,ΔC^ were kindly provided by Drs. François Doignon and Marc Crouzet [43]. The negative control plasmid pODB80-S was generated by introducing a stop codon after the *Nco*I site in frame with the Gal4-DBD ORF in pODB80 to avid extra peptide translation [48]. Bait plasmid pGBDU-RHO3^Q74L,ΔC^, pGBDU-RHO3^T30N,ΔC^ were constructed by PCR amplification of the respective *rho3* mutant without the C-terminal CAAX box using plasmids pRS426-RHO3^Q74L^ and pRS426-RHO3^T30N^ as templates and ligated into *EcoR*I- and *Sal*I-digested pGBDU-C1 (2μ, *URA3*, *GAL4-BD*) [49]. pGBDU-CDC42^Q61L,C188S^ and pGBDU-CDC42^D118A,C188S^ were generated by inserting the *EcoR*I- and *Sal*I-digested fragments from pEG202-CDC42^Q61L,C188S^ and pEG202-CDC42^D118A,C188S^ [13] into pGBDU-C1 (2μ, *URA3*, *GAL4-BD*).

For the bimolecular fluorescence complementation (BiFC) assay, plasmid pVN1 (*CEN*, *URA3*, *P*_*MET25*_ *-Venus-N-T*_*CYC1*_) and pVC1 (*CEN*, *HIS3*, *P*_*MET25*_ *-Venus-C-T*_*CYC1*_) [48] were used. An *Eco*RI-*Sal*I fragment of *RHO3* ^*Q74L*^ was inserted into pVN1, yielding pVN1-RHO3^Q74L^. pVN1-PMT-RHO3^Q74L^ was generated by replacing *P*_*MET25*_ promoter in pVN1-RHO3^Q74L^ with *P*_*MET25*_ *-PMT*. *PMT* encodes the first 11 a.a. of Rho3. pVC1-RGA1 was generated by inserting the *RGA1* ORF into *Hin*dIII- and *Sal*I-digested pVC1.

### Yeast strain construction

To generate strain YZT52 used for analysis of Rga1 phosphorylation, plasmid YIplac211-3HA-RGA1 was digested with *Apa*I and integrated at the *ura3* locus of strain YEF1002 (a *rga1Δ*::*HIS3*). Then, the *bar1*Δ::*LEU2* locus was PCR-amplified from strain JSY6 (a *bar1*Δ::*LEU2 pea2*Δ::*URA3*) (kindly supplied by Judy Shih from Ira Herskowitz lab at UCSF) and transformed into this strain, yielding YZT52.

### High-copy suppressor screen

Cells of yeast strain YEF473A carrying integrated pEGKT306-RGA1-C538 were transformed with a YEp13 (2μ, *LEU2*)-based genomic DNA library [50]. Transformants were grown on SRG-Leu-Ura plates at 30°C for 10 days. From ~500,000 transformants screened, 78 suppressors were isolated. 31 of them were determined to carry full-length *SEC9* (15×), *RHO3* (6×), *SSO1* (4×), *SSO2* (1×), *SEC1* (1×), and *SRO7* (4×). *SEC9*, *RHO3*, and *SSO1* were subcloned and confirmed to be solely responsible for the suppression. The rest of suppressor plasmids each carry multiple open reading frames without clearly defined roles in cellular morphogenesis.

### Yeast two-hybrid screen

A pOAD-cDNA library was transformed into cells of yeast strain pJ69-4A carrying pGBDU-RHO3^Q74L,ΔC^. Transformants were grown on SC-Leu-Ura-His plates supplemented with 2 mM 3-amino-1,2,4-triazole (3-AT) at 30°C for 10 days. The plates were then replica plated onto SC-Leu-Ura-Ade plates to allow the identification of candidate clones. The pOAD-prey library plasmids were retrieved and the cDNA inserts were sequenced. We screened ~200,000 transformants and isolated 153 positive clones. DNA sequencing showed that we have isolated N-terminally truncated *RGA1* (10×), *EXO70* (5×), and *MYO2* (9×). The *RGA1* clones encode a.a. 211–1007 (2×) and a.a. 598–1007 (8×). The *EXO70* clones all encode a.a. 243–623. The *MYO2* clones all encode a.a. 935–1574.

### Yeast two-hybrid assay

Cells of strain pJ69-4α carrying bait plasmids pODB80-S (2μ, *TRP1*, *GAL4-DBD*), pODB80-RHO1^Q68L,ΔC^, pODB80-RHO2^Q65L,ΔC^, pODB80-RHO3^Q74L,ΔC^, and pODB80-RHO4^Q131L,ΔC^ were mated with cells of strain pJ69-4A carrying prey plasmids pOAD (*CEN*, *LEU2*, *GAL4-AD*), pOAD-MYO2 (a.a. 935–1574), pOAD-EXO70 (a.a. 244–623), pOAD-RGA1-C211, and pOAD-RGA1-C598 on yeast extract-peptone-dextrose (YPD) plates and then replica plated onto SC-Leu-Trp plates to select for diploid cells that harbor both bait and prey plasmids. Diploid cells were replica plated onto SC-Leu-Trp-Ade plates plates and checked for growth at 30°C. In some experiments, bait plasmids pGBDU-C1 (2μ, *URA3*, *GAL4-DBD*), pGBDU-RHO3^Q74L,ΔC^, pGBDU-RHO3^T30N,ΔC^, pGBDU-CDC42^Q61L,C188S^, and pGBDU-CDC42^D118A,C188S^ as well as prey plasmids pGAD-C1 (2μ, *LEU2*, *GAL4-AD*), pGAD-RGA1 segments were also used.

### Immunoprecipitation, dephosphorylation and cell synchronization

Yeast cells were lysed in lysis buffer (10 mM HEPES-NaOH, 50 mM NaCl, 10% glycerol, 0.1% NP-40, pH7.5, plus 1×protease inhibitor cocktail and 1×phosphatase inhibitor cocktail) by vortexing in the presence of glass beads at 4°C. Cell debris was removed by centrifugation at 10,000 × g for 15 min. For immunoprecipitation, the supernatant was diluted with lysis buffer and incubated with mouse monoclonal anti-HA antibody HA.11 affinity matrix (Covance, Richmond, CA) for 2 hr at 4°C. The beads were washed three times. Bound proteins were eluted by boiling for 5 min in 2 × SDS sample buffer. Samples were then analyzed by SDS-PAGE, followed by standard immunoblotting procedure using enhanced chemiluminescence (ECL) reagents. Mouse monoclonal anti-HA and rabbit polyclonal anti-porin antibodies were used for the blotting.

For the dephosphorylation of immunoprecipitates, bound proteins were treated with 1 unit of shrimp alkaline phosphatase (SAP, USB Corporation, Cleveland, OH) in the dephosphorylation buffer (50 mM Tris-HCl, 5 mM MgCl_2_, pH 8.5, plus 1× protease inhibitor cocktail) for 1 hr at 37°C before eluted from the beads. For the synchronization of cells of strain YZT52 in G1, cells were incubated with α factor at the final concentration of 0.5 μg/ml for 120 min at 30°C. Cells were then washed and resuspended in fresh medium.

### Microscopy

An Olympus BX51 microscope (Tokyo, Japan) and a Retiga 2000R CCD camera (QImaging Corporation, Canada) were used to visualize cell morphology by differential interference contrast (DIC) and fluorescent microscopy. The images were acquired using QCapture Suite (QImaging Corporation, Canada). Image processing was performed with ImagePro Plus (Glen Mills, PA). For the examination of F-actin and DNA, yeast cells were stained with 1.0 μg/ml phalloidin-TRITC (tetramethyl rhodamine isocyanate) (Sigma-Aldrich) and 1.0 μg/ml of Hoechst 33258 (Polysciences, Inc.) at room temperature for 30 min and washed three times with 1×phosphate-buffered saline (PBS) (pH 8.0). Methylene blue staining of yeast cells was performed as described previously [23]. Methylene blue staining solution (0.001% methylene blue, 2% sodium citrate) was mixed with an equal volume of cell culture, and cells were observed immediately.
